# The contribution of executive control to semantic cognition: Convergent evidence from semantic aphasia and executive dysfunction

**DOI:** 10.1111/jnp.12142

**Published:** 2018-01-03

**Authors:** Hannah E. Thompson, Azizah Almaghyuli, Krist A. Noonan, Ohr barak, Matthew A. Lambon Ralph, Elizabeth Jefferies

**Affiliations:** ^1^ School of Psychology University of Surrey UK; ^2^ Department of Psychology and York Neuroimaging Centre University of York UK; ^3^ School of Social and Community Medicine University of Bristol UK; ^4^ Brain Injury Rehabilitation Trust (BIRT) York UK; ^5^ Neuroscience and Aphasia Research Unit Division of Neuroscience and Experimental Psychology School of Biological Sciences University of Manchester UK

**Keywords:** aphasia, control, executive dysfunction, semantic

## Abstract

Semantic cognition, as described by the controlled semantic cognition (CSC) framework (Rogers *et al*., 2015, *Neuropsychologia*, 76, 220), involves two key components: activation of coherent, generalizable concepts within a heteromodal ‘hub’ in combination with modality‐specific features (spokes), and a constraining mechanism that manipulates and gates this knowledge to generate time‐ and task‐appropriate behaviour. Executive–semantic goal representations, largely supported by executive regions such as frontal and parietal cortex, are thought to allow the generation of non‐dominant aspects of knowledge when these are appropriate for the task or context. Semantic aphasia (SA) patients have executive–semantic deficits, and these are correlated with general executive impairment. If the CSC proposal is correct, patients with executive impairment should not only exhibit impaired semantic cognition, but should also show characteristics that align with those observed in SA. This possibility remains largely untested, as patients selected on the basis that they show executive impairment (i.e., with ‘dysexecutive syndrome’) have not been extensively tested on tasks tapping semantic control and have not been previously compared with SA cases. We explored conceptual processing in 12 patients showing symptoms consistent with dysexecutive syndrome (DYS) and 24 SA patients, using a range of multimodal semantic assessments which manipulated control demands. Patients with executive impairments, despite not being selected to show semantic impairments, nevertheless showed parallel patterns to SA cases. They showed strong effects of distractor strength, cues and miscues, and probe–target distance, plus minimal effects of word frequency on comprehension (unlike semantic dementia patients with degradation of conceptual knowledge). This supports a component process account of semantic cognition in which retrieval is shaped by control processes, and confirms that deficits in SA patients reflect difficulty controlling semantic retrieval.

## Background

Successful retrieval of semantic knowledge in a context‐specific and timely manner depends on the interaction of multiple processes, including (1) the conversion of sensory input into meaning (Sharp, Scott, & Wise, 2004), (2) generalizable conceptual representations (Lambon Ralph, Sage, Jones, & Mayberry, 2010; Patterson, Nestor, & Rogers, 2007), and (3) flexible control over the retrieval of knowledge, such that semantic processing focuses on information appropriate to the context even when this is not necessarily the dominant feature or association (Jefferies & Lambon Ralph, 2006). An interaction between these components is envisaged within the controlled semantic cognition (CSC) framework (Jefferies, 2013; Lambon Ralph, Jefferies, Patterson, & Rogers, 2017; Rogers *et al*., 2015). Patients with damage to these different components of semantic cognition show qualitatively different patterns of semantic impairment (Corbett, Jefferies, Burns, & Lambon Ralph, 2014; Corbett, Jefferies, Ehsan, & Lambon Ralph, 2009; Jefferies, Hoffman, Jones, & Lambon Ralph, 2008; Jefferies & Lambon Ralph, 2006; Jefferies, Patterson, & Lambon Ralph, 2008; Jefferies, Rogers, Hopper, & Lambon Ralph, 2010). However, the mechanisms underpinning semantic control are underspecified; in particular, it is unclear the extent to which this capacity draws on domain‐general executive control, and whether there are control processes specific to the semantic domain which allow the interaction of stored representations and current semantic contexts (Davey *et al*., 2016; Noonan, Jefferies, Visser, & Lambon Ralph, 2013). By comparing patients with deficits of stored knowledge with deregulated semantic control and executive dysfunction, this study aimed to elucidate these mechanisms.

The differentiation of the representation versus control components of semantic cognition can be observed in contrastive neuropsychological and neuroscience data. Patients with semantic dementia (SD) have focal atrophy within bilateral anterior and ventral parts of temporal lobe (ATL) (Binney, Embleton, Jefferies, Parker, & Lambon Ralph, 2010; Mion *et al*., 2010; Mummery *et al*., 2000) and display a gradual degradation of semantic knowledge, such that information about specific entities and less familiar items is lost first (Hodges, Graham, & Patterson, 1995; Hodges, Patterson, Oxbury, & Funnell, 1992; Lambon Ralph & Patterson, 2003; Patterson *et al*., 2007). This pattern, along with converging evidence from neuroimaging and neurostimulation studies (Binney *et al*., 2010; Pobric, Jefferies, & Lambon Ralph, 2010; Rice, Lambon Ralph, & Hoffman, 2015; Visser & Lambon Ralph, 2011), suggests that the ATL acts as a transmodal conceptual ‘hub’, supporting the representation of the meanings of words, objects, faces, and sounds (Bozeat, Lambon Ralph, Patterson, Garrard, & Hodges, 2000; Patterson *et al*., 2006, 2007). In contrast, semantic aphasia (SA) patients have a deficit characterized by inconsistent semantic ‘access’ (Warrington & Cipolotti, 1996) and have greater deficits under circumstances where control demands are high, suggesting they have intact semantic representations but are unable to focus conceptual processing on currently relevant features and associations in the absence of external constraints (Jefferies & Lambon Ralph, 2006). The term ‘semantic aphasia’ transcends classical ‘Boston’ aphasia classifications, and patients can present different aphasia profiles as their spoken language skills are variable. SA patients have left‐hemisphere damage focused on inferior frontal gyrus (IFG) and posterior middle temporal gyrus (pMTG) (Gardner *et al*., 2012; Noonan, Jefferies, Corbett, & Lambon Ralph, 2010; Thompson, Robson, Lambon Ralph, & Jefferies, 2015), and deficits affecting the comprehension of words, objects, environmental sounds, and actions (Corbett, Jefferies, Ehsan, *et al*., 2009; Corbett, Jefferies, & Lambon Ralph, 2009, 2011; Gardner *et al*., 2012; Jefferies & Lambon Ralph, 2006). Similar brain regions have been implicated in the control of semantic processing by neuroimaging and neurostimulation studies (Badre, Poldrack, Paré‐Blagoev, Insler, & Wagner, 2005; Davey, Cornelissen, *et al*., 2015; Davey, Rueschemeyer, *et al*., 2015; Davey, Thompson, *et al*., 2016; Krieger‐Redwood, Teige, Davey, Hymers, & Jefferies, 2015; Noonan *et al*., 2013; Thompson‐Schill, D'Esposito, Aguirre, & Farah, 1997; Whitney, Kirk, O'Sullivan, Lambon Ralph, & Jefferies, 2011). These regions include dorsal and posterior IFG and inferior frontal sulcus (IFS) within the multidemand executive network (Duncan, 2010; Humphreys & Lambon Ralph, 2015) – that is, regions implicated in executive control across different domains and not just semantic tasks – as well as anterior IFG and pMTG more specifically implicated in semantic control. As a consequence, SA patients are likely to have deficits of both semantic control and domain‐general executive control (Jefferies & Lambon Ralph, 2006).

The current study examined the relationship between semantic control and domain‐general executive processes by examining patients with executive dysfunction (i.e., patients who met the criteria for ‘DYS’). These patients were *not* selected on the basis of their performance on semantic tasks, unlike cases with SA. If domain‐general executive processes interact with semantic representations to support controlled aspects of semantic cognition, then SA and DYS cases should both show sensitivity to semantic control manipulations in the context of intact knowledge: They may have qualitatively similar semantic deficits which contrast with the impairment in SD. In line with this prediction, neuroimaging studies of healthy participants have shown that tasks manipulating the control demands of semantic tasks recruit regions within the multidemand network (Humphreys & Lambon Ralph, 2015) – including IFS, intraparietal sulcus, and pre‐SMA (Badre *et al*., 2005; Humphreys & Lambon Ralph, 2015; Noonan *et al*., 2013; Thompson‐Schill *et al*., 1997). These areas are thought to sustain top‐down constraints supporting goal‐driven aspects of cognition across domains (Duncan, 2010, 2013; Fedorenko, Duncan, & Kanwisher, 2013). Top‐down control is likely to be necessary for many semantic tasks (Whitney, Kirk, O'Sullivan, Lambon Ralph, & Jefferies, 2012) – for example, when a target concept specified by the instructions is encountered alongside strong distractors. Both DYS and SA groups are expected to show deficits on these types of tasks.

There are also situations in which the information that is retrieved about a concept has to be shaped according to the semantic context, in the absence of an explicit goal. An example is comprehending an ambiguous word such as ‘ash’, which depends on the context (e.g., ‘the beech and the ash were common in the local forests’; Rodd, Davis, & Johnsrude, 2005). Brain regions that support this process are potentially unique to the semantic domain. An activation likelihood estimation meta‐analysis examining the neural basis of semantic control highlighted extensive overlap between sites implicated in controlled semantic retrieval and cognitive control more broadly, but also some regions outside the multidemand network (Humphreys & Lambon Ralph, 2015; Noonan *et al*., 2013): In particular, ventral and anterior LIFG (pars orbitalis) and posterior middle temporal gyrus (pMTG) respond to diverse manipulations of semantic control, but not to challenging non‐semantic tasks (Devlin, Matthews, & Rushworth, 2003; Gold & Buckner, 2002; Gold *et al*., 2006; Snyder, Feigenson, & Thompson‐Schill, 2007). However, given the nature of stroke and brain injury lesions, it is unlikely that there will be a clear dissociation between semantic and domain‐general executive control.

We recruited patients with evidence of executive dysfunction in their planning, reasoning, abstract thinking, cognitive flexibility, and behavioural control. Such patients are sometimes referred to using the umbrella term ‘DYS’ (Baddeley & Wilson, 1988; Wilson, Evans, Emslie, Alderman, & Burgess, 1998), although they are heterogeneous. We evaluated the semantic performance of a group of DYS patients selected on the basis of their executive and not their semantic scores and compared them to SA cases (selected to show multimodal semantic impairment) on semantic and non‐semantic assessments with varying control demands and across modalities. We also compared these two groups to patients with SD, where data were available. This case‐series comparison allows us to establish whether patients with executive dysfunction can show deficits in control‐demanding semantic tasks; and whether SA and DYS patients are similar to each other and, as previously demonstrated for SA cases, qualitatively different from patients with SD who show degradation of conceptual information. Given DYS cases have disruption to executive mechanisms, we predicted this would be reflected in performance on semantic tasks, including (1) no effect of frequency/familiarity on comprehension, (2) little correlation between semantic tasks differing in their control demands even when these include the same concepts, (3) performance related to the executive demands of each trial, such as the strength of distractors, (4) high susceptibility to being aided by cues and misled by miscues that are related to the target, and (5) correlation between semantic and executive performance.

## Method and Results

### Participants

#### DYS group

Twelve DYS patients were recruited from rehabilitation and head injury support units in York, Leeds, and Manchester, UK (see Table 1). All patients had chronic impairment from acquired brain injury sustained as an adult at least 1 year prior to testing. Patients included in this group were invited to take part in the study on the basis of their executive dysfunction, following evaluation by a clinical neuropsychologist. All patients completed the Behavioural Assessment of Dysexecutive Syndrome (BADS; Wilson, Alderman, Burgess, Emslie, & Evans, 1996) and showed poor overall performance, including scoring at least 1.5 *SD* below the expected level on at least one subscale, shown in Table 1. They were not selected on the basis of their performance on semantic tasks.

**Table 1:** Demographic information for dysexecutive patients

Patient	Age	Education	Neuroimaging and aetiology	Rule shift	Action programme	Key search	Temporal judgement	Zoo map	Six elements	BADS total	Standardized score	Classification
1	40	14	L frontotemporal damage following external insult by sharp object	2*	3*	0*	1*	1	0*	7	38	Impaired
2	38	15	Bilateral ischaemic encephalopathy of basal ganglia following hypoglycaemia	3	3*	1	1*	1	0*	9	49	Impaired
3	64	Dip	Hypoxic episode following cardiac arrest	4	2*	0*	1*	1	1*	9	53	Impaired
4	25	18	Road traffic accident (RTA)	3	4	0*	2	1	1*	11	59	Impaired
5	52	18	Bilateral anterior cerebral artery infarcts	1*	3*	1	2	1	3	11	63	Impaired
6	22	16	R frontal + L parietal damage from RTA	4	3*	1	3	0*	1*	12	65	Impaired
7	21	18	Diffuse axonal injury with small intraventricular changes following RTA	4	4	0*	2	2	1*	13	70	Borderline
8	22	16	L frontal–parietal tumour resection	4	0*	4	3	1	1*	13	70	Borderline
9	45	16	L temporal lobectomy following temporal lobe abscess	3	4	1	2	1	2*	13	73	Borderline
10	59	16	L+R frontal–parietal damage from RTA	2*	4	2	3	0*	3	14	78	Borderline
11	25	15	Enlargement of R lateral ventricle + contusions in the cerebellum and cerebrum following RTA	1*	4	3	3	3	1*	15	81	Low average
12	28	14	White‐matter damage in L PFC + R parietal contusion following violent assault	4	4	4	0*	2	2*	16	86	Low average
			Control mean (*SD*)	3.72 (0.65)	3.79 (0.41)	2.77 (1.23)	2.34 (0.81)	2.28 (1.36)	3.41 (0.91)			

Note

Patients are arranged in order of Behavioural Assessment of Dysexecutive Syndrome scores (BADS; Wilson *et al*., 1996). All subtests and control data are from the BADS. Each subtest has a score out of 4. Weak performance, at least 1.5 *SD* below the expected performance given age and educational status, is marked with *. Education = age of leaving education. Dip = postgraduate diploma. Neuroimaging summaries are based on written reports of clinical scans, except in the case of JG, CR, and GR where they were based on visual inspection of CT scans.

#### SA group

We contrasted the DYS group with performance in 24 SA patients, most of whom had participated in our previous investigations of semantic control deficits (see Table 2; Almaghyuli, Thompson, Lambon Ralph, & Jefferies, 2012; Jefferies & Lambon Ralph, 2006; Thompson, Henshall, & Jefferies, 2016; Thompson *et al*., 2015). SA patients had a cerebrovascular accident at least a year previously. Patients were selected for inclusion if they exhibited multimodal semantic deficits (poor performance on the word and picture versions of the Camel and Cactus Test (CCT) (Bozeat *et al*., 2000)). Although all the SA patients showed the hallmarks of semantic control impairment, they were not selected on this basis. Moreover, as the patient group was defined using test scores and not lesion location, there was some variability in the areas of damage, as described in Table 2. SA patients typically have damage to left frontal and/or temporoparietal regions (Corbett *et al*., 2011; Gardner *et al*., 2012; Noonan *et al*., 2010), and importantly, none had damage to ventral parts of ATL, as this is a watershed region (Phan, Donnan, Wright, & Reutens, 2005; Phan, Fong, Donnan, & Reutens, 2007). Unsurprisingly, SA patients with a stroke aetiology were significantly older than DYS patients with brain injury: *t* (27) = 6.315, *p* < .001. There was, however, no difference in education level (*t* < 1).

**Table 2:** Aphasia classifications and neuroimaging summaries for the SA participants

Patient	Age	Edu	Neuroimaging summary	Aphasia type	BDAE comprehension	BDAE fluency	Word repetition (%)	Cookie theft (words per min)
CH	NA	NA	No scan	Mixed transcortical	NT	NT	NT	15
BK	65	NA	L frontal–temporal–parietal	Broca's	NT	NT	94	12
HN	80	15	L occipital–temporal	Anomic/TSA	NT	NT	86	59
SC	80	16	L occipital–temporal (+ small R frontal infarct)	Anomic/TSA	37	90	98	84
KS	59	16	L temporal	TSA	NT	NT	94	84
EW	74	15	L occipital–temporal	TSA	NT	NT	80	NT
MD	88	NA	L frontal	TSA	NT	NT	NT	46
DB	76	16	L frontal–temporal–parietal	TSA	13	90	85	11
MP	NA	NA	L frontal–temporal–parietal	Global	NT	NT	53	0
PG	63	18	L frontal & capsular	TSA	20	40	91	27
KH	73	14	L frontal–parietal–occipitotemporal	Mixed transcortical	30	30	80	29
PH	75	15	L frontal–temporal	Anomic	NT	NT	NT	18
JD	81	16	Compression of L lateral ventricle & capsular	Mixed transcortical	NT	NT	93	NT
KA	78	14	L frontal–parietal	Global	0	23	0	NT
GH	56	18	L frontal–parietal	Global	NT	NT	NT	3
NY	67	15	L frontal–parietal	Conduction	47	37	81	42
MS	73	14	No scan	Global	10	0	0	NT
BB	59	16	L frontal	Mixed transcortical	10	17	96	11
EG	59	18	L frontal–temporal	Global	NT	NT	NT	0
MJ	NA	NA	No scan	Mixed transcortical	NT	NT	35	21
ME	40	16	L occipital–temporal	TSA	33	100	100	63
JM	69	18	L frontal–parietal	TSA	22	63	95	26
EC	71	16	L frontal–parietal	Global	NT	NT	16	0
LS	75	15	L frontal–parietal–occipitotemporal	TSA	13	90	96	30

Notes

NA = data not available; NT = not tested.

Patients are arranged in order of semantic performance, taking the average scores from 96‐item Synonym Judgement Task, CCTw, and CCTp – where scores were unavailable for a particular task, the average of the remaining tasks was taken. BDAE = Boston Diagnostic Aphasia Examination (Goodglass & Kaplan, 1983). BDAE Comprehension score is a percentile derived from three subtests (word discrimination, commands, and complex ideational material). BDAE Fluency percentile is derived from phrase length, melodic line, and grammatical form ratings. BDAE Repetition percentile is average of word and sentence repetition. TSA (transcortical sensory aphasia) was defined as good or intermediate fluency/repetition and poorer comprehension. Word/non‐word repetition: tests 8 and 9 from PALPA (Psycholinguistic Assessments of Language Processing in Aphasia; Kay, Lesser, & Coltheart, 1992).

#### SD patients

Where possible, we compared DYS and SA patients with data from SD patients. This allowed us to assess whether both DYS and SA groups showed a dissociation from the degradation of semantic representations seen in SD, in line with the findings of Jefferies and Lambon Ralph (2006). The Cambridge Multimodal Semantic Battery was examined in 10 SD cases first reported in Bozeat *et al*. (2000). For the 96‐item Synonym Judgement Task, data for 11 SD patients were available from Jefferies, Patterson, Jones, and Lambon Ralph (2009). Eight SD patients tested in Manchester and Bath were available for the semantic distance task: GE, TM, JW, NH, JA, BS, ET, and PW. They have all been previously described (Corbett *et al*., 2014; Hoffman, Evans, & Lambon Ralph, 2014; Hoffman, Jones, & Lambon Ralph, 2012; Hoffman & Lambon Ralph, 2011; Jefferies, Hoffman, *et al*., 2008). All SD patients fulfilled published diagnostic criteria (Hodges *et al*., 1992): They had word‐finding difficulties in the context of fluent speech and showed impaired semantic knowledge and single‐word comprehension: In contrast, phonology, syntax, visual–spatial abilities, and day‐to‐day memory were relatively well preserved. Structural MRI revealed focal atrophy involving the inferolateral regions of bilateral temporal lobes in every case.

### Background neuropsychological assessments

The SA and DYS patients were examined on a range of standard tests to assess executive function and semantic performance: (1) *Brixton Spatial Anticipation Task* (BSAT; Burgess & Shallice, 1997), in which the participants predict the location of a moving dot in a spatial display; (2) *Raven's Coloured Progressive Matrices* (RCPM; Raven, 1962), a nonverbal reasoning task in which participants identify which of six missing elements complete a spatial pattern; (3) Digit span, forwards and backwards (Wechsler, 1987); (4) Letter fluency, which requires participants to produce as many words as possible within 1 min which begin with a certain letter (F, A, S); (5) 64‐item Cambridge Semantic Battery (Bozeat *et al*., 2000), which presented the same items in multiple tasks: (i) spoken word–picture matching (WPM), (ii) picture naming, (iii) picture CCT, and (iv) word CCT – the CCT involved identifying which of four pictures/words was most associated with a probe picture/word (e.g., camel with cactus, rose, tree, or sunflower?); (6) 48‐item Environmental Sounds Task (Bozeat *et al*., 2000), which included (i) matching environmental sounds (e.g., a dog barking) to pictures (S‐P) and (ii) matching spoken words to pictures (W‐P); and (7) 96‐item Synonym Judgement Task (Jefferies *et al*., 2009) split into low‐ or high‐frequency and low‐, medium‐, and high‐imageability trials. A probe word was matched to a synonym target presented with two unrelated distractors. The words were printed and also read aloud to the participants.

#### Results

Dysexecutive syndrome background performance is displayed in Table 3 and SA and SD data in Table S1. DYS patients performed at a higher level than SA patients on both semantic and executive tasks. There was a significant group difference on all executive and semantic tasks (*p* ≤ .045) except the Brixton, where there was a trend towards a group difference: *t* (32) = 1.876, *p* = .070.

**Table 3:** Performance across background tests – DYS patients

	Brixton	Ravens	Digit span	Backwards digit span	Letter fluency (F, A, S)	WPM	Naming	CCTw	CCTp	S‐P env sounds	W‐P env sounds	96‐item synonym	Low imageability	Medium imageability	High imageability	Low frequency	High frequency
Max	54	36	8	7	–	64	64	64	64	48	48	96	32	32	32	48	48
Normal average		32.9^a^	6.8^a^	5.6^a^	44.2	63.7	62.3	61	59	41	48	94.5	30.8	32	31.9	47.4	47.1
*SD*		2.4	0.6	1	11.2	0.5	1.6	2.1	3.1	2.5	0.6	1.8	1.3	0.7	0.5	1	1
Cut‐off	28	28	5.6	2	21.8	62.7	59.1	57	53	36	47	89	27.6	30.8	30.9	44.9	44.4
Average DYS	27.8	28	5.5	3.6	22.9	60.7	56	51.4	51.7	35.3	46.1	77.7	21.7	26.5	29.5	38	39.7
Average SA	21.6	22.6	4	1.9	6.7	52.1	28.9	39.1	40	26.8	35.9	64.9	16.8	22.3	26.8	32.7	33
Average SD	–	–	6.5	4.5	20.9	45.5	26.5	37.5	41.2	21.6	33.1	61.5	15.7	21.4	24.4	23.5	37.9
1	6	23***	5*	3*	5***	54***	49***	42***	47**	37	44***	77***	26**	26***	25***	36***	41***
2	13	22***	4***	3*	22	60***	60	49**	56	35	46*	74***	20***	27***	27***	39***	35***
3	37	27*	8	6	38	64	64	55*	43**	35	48	92	31	29**	32	45*	47
4	31	32	7	4	33	63	63	48***	49*	39	46*	72***	18***	23***	31	39***	33***
5	26	27*	5*	3*	34	57***	61	49**	50*	41	47	82***	21***	29**	32	36***	46
6	18	31	6	3*	5***	60***	56**	52**	55	30**	46*	75***	21***	24***	30**	38***	37***
7	40	28	4***	3*	9***	62*	63	58	55	35	46*	75***	21***	26***	28***	31***	44*
8	17	30	5*	3*	17**	63	61	50**	55	36	47	77***	23***	26***	28***	38***	39***
9	28	29	4***	3*	4***	60***	21***	51**	59	32*	45**	69***	17***	22***	30**	38***	31***
10	31	26*	6	4	29	63	58*	58	53	33*	46*	83***	26**	27***	30**	41***	42***
11	41	29	6	4	41	59***	55**	50**	48*	37	45**	78***	20***	29**	29***	40***	38***
12	45	30	6	4	38	63	62	55*	50*	34*	47	78***	16***	30*	32	35***	43**

*p < .05; **p < .01; ***p < .001, two‐tailed probability using the ‘Singlims’ procedure (Crawford & Garthwaite, 2002), which uses a modified *t*‐statistic to examine whether an individual is significantly below a control group, taking into account group size and standard deviation.

^a^Norms from healthy controls tested at the University of York, number of controls as follows: Ravens = 20; digit span = 17; and backwards digit span = 10. Brixton = Brixton Spatial Rule Assessment (Burgess & Shallice, 1997); Ravens = Ravens Coloured Progressive Matrices (Raven, 1962); WPM = word–picture matching, naming; CCTw = Camel and Cactus Test, words; CCTp = Camel and Cactus Test, pictures. All four tasks (CCTw, CCTp, naming, and WPM) are from the Cambridge Semantic Battery (Bozeat *et al*., 2000). S‐P env sounds = sound–picture matching; W‐P env sounds = WPM, from the Environmental Sounds Task (Bozeat *et al*., 2000). Ninety‐six‐item Synonym Judgement Task and the subscores according to imageability/frequency (Jefferies *et al*., 2009). Averaged data are presented for SA and SD; data used for these groups are displayed in Table S1.

### Analysis of covariance of semantic and executive impairment

**Figure 1:**
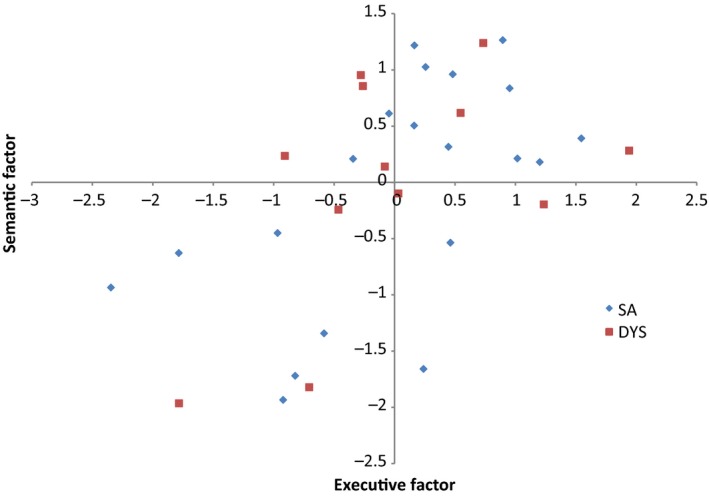
Correlation between executive and semantic performance for DYS and SA patients. [Colour figure can be viewed at http://www.wileyonlinelibrary.com]

Patients who have damage to regions implicated in ‘accessing’ semantic information have been shown to have deficits beyond semantic cognition, in domain‐general executive control (Jefferies & Lambon Ralph, 2006). Therefore, we predicted that SA patients with semantic deficits would show some degree of executive dysfunction, while DYS cases with executive impairment would show correlated semantic deficits. Factor analysis was used to extract a single factor across multiple tasks which tapped the same concept (e.g., semantic, executive), for the patients in each group separately. The ‘executive’ scores included Brixton and Raven's Coloured Progressive Matrices. The ‘semantic’ scores included WPM, CCTw, CCTp, and the 96‐item Synonym Judgement Task. These tasks where chosen to not require a spoken response (which in some SA participants would result in low scores linked to deficits in speech production, unduly influencing the group comparison; see Appendix S1). The factor scores reflected the performance of each patient, relative to others in their group, and thus allowed us to establish whether patients with greater executive deficits also had more severe semantic impairment within each group (shown in full in Table S2). In ANCOVA on mean‐centred scores for each group, there was a strong effect of executive impairment on semantic performance: *F* (1, 28) = 11.230, *p* = .002, but no main effect of group (*F* < 1) or interaction (*F* < 1). This is shown in Figure 1.

#### Analysis of covariance within the semantic battery

The Cambridge Semantic Battery probes the same items across four tests: CCT of semantic association for words and pictures, WPM, and picture naming. Additionally, the Environmental Sounds Task assesses performance on the same items across word–picture and sound–picture matching (Bozeat *et al*., 2000). Previous research suggests that SD patients with degraded semantic representations show high correlations across all task pairs (Bozeat *et al*., 2000; Jefferies & Lambon Ralph, 2006). In contrast, SA patients only show correlations in performance where control demands are matched (e.g., CCT words vs. CCT pictures); they are more inconsistent when tasks with different demands are compared (e.g., CCT words vs. WPM or naming). ANCOVA was used to examine differences between groups in the consistency of performance across pairs of tasks (see Table 4). Where SD cases were in the analyses (SA vs. SD or DYS vs. SD), there were more significant predictive effects, and more interactions with group (reflecting that the predicting performance from one task to another stemmed from the SD group). There was strong similarity between SA and DYS patients and no significant group‐by‐consistency interactions. In contrast, comparisons of SD patients with SA or DYS patients revealed numerous group‐by‐task interactions, reflecting more consistent performance in the SD group.

**Table 4:** ANCOVAs assessing predictability of performance on one semantic task compared with another across groups

	DV	Covariate	Covariate *F*‐value	Group *F*‐value	Covariate × group
DYS and SA	CCTp	CCTw	2.38	1.11	0.80
	CCTp	WPM	0.11	0.09	0.01
	CCTp	Naming	0.11	5.96*	4.02
	CCTw	CCTp	2.23	1.06	0.65
	CCTw	WPM	3.97	0.36	0.58
	CCTw	Naming	3.76	1.98	1.11
	WPM	CCTp	0.16	0.22	0.07
	WPM	CCTw	3.32	0.01	0.00
	WPM	Naming	3.73	1.21	1.10
	Naming	CCTp	0.03	5.23*	3.86
	Naming	CCTw	2.87	0.64	0.29
	Naming	WPM	2.55	0.00	0.02
	S‐P	W‐P	0.03	0.00	0.02
	W‐P	S‐P	0.01	0.12	0.00
DYS and SD	CCTp	CCTw	6.96*	2.14	2.73
	CCTp	WPM	2.54	1.43	1.45
	CCTp	Naming	5.39*	15.31*	18.74*
	CCTw	CCTp	10.88*	7.67*	5.93*
	CCTw	WPM	12.61*	0.02	0.10
	CCTw	Naming	7.61*	4.50*	4.06
	WPM	CCTp	13.28*	12.72*	11.61*
	WPM	CCTw	14.56*	0.88	1.19
	WPM	Naming	11.38*	5.53*	7.01*
	Naming	CCTp	0.03	13.33*	10.33*
	Naming	CCTw	3.05	0.43	0.18
	Naming	WPM	3.29	0.02	0.13
	S‐P	W‐P	0.37	0.15	0.08
	W‐P	S‐P	3.38	3.03	3.04
SA and SD	CCTp	CCTw	31.36*	0.06	0.16
	CCTp	WPM	12.86*	2.17	3.75
	CCTp	Naming	26.95*	1.26	3.61
	CCTw	CCTp	40.56*	6.10*	4.36*
	CCTw	WPM	25.10*	2.45	2.36
	CCTw	Naming	22.49*	2.68	2.17
	WPM	CCTp	25.35*	17.14*	13.44*
	WPM	CCTw	24.04*	1.49	1.79
	WPM	Naming	33.22*	8.69*	5.88*
	Naming	CCTp	21.51*	0.65	0.48
	Naming	CCTw	18.92*	0.35	0.13
	Naming	WPM	23.06*	0.55	0.26
	S‐P	W‐P	7.46*	9.87*	7.04*
	W‐P	S‐P	3.18	2.45	2.82

CCTw = Camel and Cactus words; CCTp = Camel and Cactus pictures; WPM = word–picture matching.

All from the Cambridge Semantic Battery (Bozeat *et al*., 2000). W‐P and S‐P are word–picture and sound–picture matching tasks from the Environmental Sounds Task (Bozeat *et al*., 2000). Each line represents a separate analysis. In each analysis, we assessed the value of one task (the DV) in relation to the group and while controlling for the influence of performance on another task (the covariate). Significant covariate results suggest an effect of task performance influencing performance on another task (the DV). Where this interacts significantly with group, this suggests a difference in the influence of this covariate between the groups. Values presented are the *F*‐statistics.

a *p* ≤ .05.

### Summary: ANCOVA

Dysexecutive syndrome and SA patients showed an equivalent relationship between executive and semantic performance and did not differ in the ability of one semantic task to predict performance on another task. In contrast, SD patients were more consistent in their response to the same item across tasks with different control demands.

### Effects of familiarity and frequency on performance

A processing benefit for items that are high in familiarity and frequency is commonly found in healthy subjects. In SD patients, this pattern is typically exaggerated: There are more learning episodes for high‐frequency concepts, giving rise to stronger semantic representations, and these items are encountered more often as the semantic system degenerates, which may have a protective effect (Jefferies, Rogers, & Lambon Ralph, 2011; Rogers *et al*., 2004; Rogers, Patterson, Jefferies, & Lambon Ralph, 2015). In contrast, SA patients can show absent (or reverse) frequency effects. Semantic judgements involving high‐frequency targets and distractors are thought to require additional control, as these items appear in multiple ‘diverse’ contexts and their meaning out of context is more ambiguous (Almaghyuli *et al*., 2012; Hoffman, Rogers, & Lambon Ralph, 2011). Therefore, individuals with executive dysfunction are also expected to show absent or reverse frequency effects.

#### Ninety‐six‐item Synonym Judgement Task

**Figure 2:**
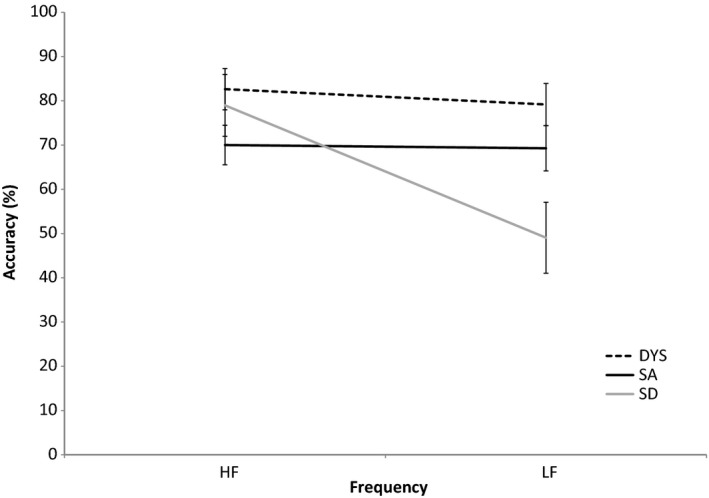
Performance on the 96‐item Synonym Judgement Task for high‐ and low‐frequency items across patient groups. Error bars show standard error of mean.

The effects of frequency (high, low) and imageability (high, medium, low) on synonym judgement performance were compared across three groups (SD, SA, and DYS). There was a main effect of group: *F* (2, 40) = 4.758, *p* = .014; frequency: *F* (1, 40) = 39.238, *p* < .001; and imageability: *F* (2, 80) = 96.776, *p* < .001. Imageability did not interact with group (*F* < 1). Frequency interacted with group: *F* (2, 40) = 24.748, *p* < .001, reflecting no difference between high‐ and low‐frequency performance in DYS or SA (*t* < 1), but a significant effect in SD: *t* (10) = 8.116, *p* < .001, in line with our predictions. This pattern is shown in Figure 2. In addition, there was a frequency‐by‐imageability interaction: *F* (2, 80) = 5.213, *p* = .007, but no three‐way interaction with group.

#### Cambridge semantic battery

Ratings of familiarity for these items were taken from Garrard, Lambon Ralph, Hodges, and Patterson (2001). Using a median split of familiarity rating (‘high’ vs. ‘low’), univariate analysis examined the effects of familiarity on accuracy per group and task, including all tasks in the Cambridge Semantic Battery and Environmental Sounds Task, shown in Table S3 and Figure S1. We then used logistic regression to analyse the effects of familiarity on the four tasks in the Cambridge Semantic Battery (CCTp, CCTw, WPM, and naming) across groups. Variables entered into the model to predict accuracy included familiarity, group, familiarity x group, item, patient ID, and task. We ran the same regression analysis including all groups, and then each pair of groups. The interaction between familiarity and group was only significant for analyses which included the SD group (see Table 5), extending the findings originally reported in Jefferies and Lambon Ralph (2006). The SD patients showed strong and significant effects of familiarity on all tasks. The SA and DYS cases showed weaker effects of familiarity, supporting our hypothesis.

**Table 5:** Effects of familiarity on performance at the Cambridge Semantic Battery

	All groups (SA, SD, DYS)	SA & DYS	SA & SD	DYS & SD
Familiarity	n.s.	n.s.	n.s.	n.s.
Group	n.s.	n.s.	n.s.	n.s.
Familiarity × group	*W* = 43.473, *p* < .001	n.s.	*W* = 41.926, *p* < .001	*W* = 10.469, *p* = .001
Item	*W* = 609.055, *p* < .001	*W* = 496.979, *p* < .001	*W* = 464.334, *p* < .001	*W* = 337.052, *p* < .001
Patient ID	*W* = 1,212.089, *p* < .001	*W* = 650.095, *p* < .001	*W* = 1,122.514, *p* < .001	*W* = 651.861, *p* < .001
Task	*W* = 584.624, *p* < .001	*W* = 423.398, *p* < .001	*W* = 558.022, *p* < .001	*W* = 218.744, *p* < .001

n.s. = not significant.

Four separate logistic regression analyses were conducted of Cambridge Semantic Battery tasks: Camel and Cactus words and pictures, word–picture matching, and picture naming (Bozeat *et al*., 2000). Variables entered into the model: familiarity, group, familiarity x group, item, patient ID, and task. *p* values reported if *p* < .1.

#### Environmental sounds task

Logistic regression was also used to analyse the effect of familiarity on accuracy on the Environmental Sounds Tasks (Bozeat *et al*., 2000). Variables entered into the model to predict accuracy included familiarity, group, familiarity x group, item, patient ID, and task (word–picture and sound–picture matching). We ran the same regression analysis including all groups, and then each pair of groups (shown in Table 6). SD patients were strongly affected by familiarity in both tasks, while SA patients did not show an effect of familiarity in either task. In this analysis, DYS patients showed a familiarity effect in the sound but not the word task.

**Table 6:** Effects of familiarity on performance at the Environmental Sounds Task

	All groups (SA, SD, DYS)	SA, DYS	SA, SD	DYS, SD
Familiarity	*W* = 21.768, *p* < .001	*W* = 3.911, *p* = .048	*W* = 13.915, *p* < .001	*W* = 13.016, *p* < .001
Group	n.s.	n.s.	n.s.	n.s.
Familiarity × group	*W* = 36.722, *p* < .001	*W* = 16.170, *p* < .001	*W* = 30.205, *p* < .001	n.s.
Item	*W* = 289.778, *p* < .001	*W* = 227.280, *p* < .001	*W* = 213.823, *p* < .001	*W* = 181.217, *p* < .001
Patient ID	*W* = 220.781, *p* < .001	*W* = 97.615, *p* < .001	*W* = 203.730, *p* < .001	*W* = 144.581, *p* < .001
Task	*W* = 211.108, *p* < .001	*W* = 160.721, *p* < .001	*W* = 112.467, *p* < .001	*W* = 157.033, *p* < .001

n.s. = not significant.

Logistic regression of Environmental Sounds Tasks: sound–picture matching and word–picture matching (Bozeat *et al*., 2000). Variables entered into the model: familiarity, group, familiarity x group, item, patient ID, and task. *p* values reported if *p* < .1.

### Summary: Effects of familiarity and frequency

Semantic aphasia and DYS patients showed little effect of frequency/familiarity, in contrast to strong effects of this variable on SD performance (with the exception of the Environmental Sounds Task, in which DYS patients showed a familiarity effect). Executive dysfunction may disproportionately disrupt performance on high‐frequency/familiarity concepts as there are many possible associations to these items, and this is thought to increase their control demands.

### Factors affecting difficulty in the Camel and Cactus Test

Here, we assessed the effects of two aspects of difficulty on semantic judgements: (1) the co‐occurrence of the probe and target and (2) the rated ease of rejecting the distractors. We predicted that the frequency of co‐occurrence of the probe and target would affect all patient groups. SD patients with degraded semantic knowledge are highly sensitive to frequency effects in general (Bird, Lambon Ralph, Patterson, & Hodges, 2000; Jefferies *et al*., 2009): The relationship between probe–target pairs that occur together rarely may be encoded weakly in ATL, and therefore, this information may be relatively vulnerable to damage. Decisions about infrequently co‐occurring probes and targets are also thought to have higher control demands (Jefferies & Lambon Ralph, 2006; Noonan *et al*., 2010). For strong but not weak associations, unconstrained interactive activation should rapidly identify the target: Consequently, SA and DYS cases are expected to show poorer retrieval of more unusual probe–target pairs.

In contrast, the ease of rejecting the distractors was predicted to differentiate the groups. Patients with executive deficits should find it more difficult to suppress strong distractors, which create competition with the target. We predicted that SA and DYS patients should respond similarly to this factor, with poorer performance on trials where the distractors were harder to ignore. SD patients were not expected to be as sensitive to the strength of distractors, because the semantic relationships that normally make distractors potent are eroded in SD.

**Figure 3:**
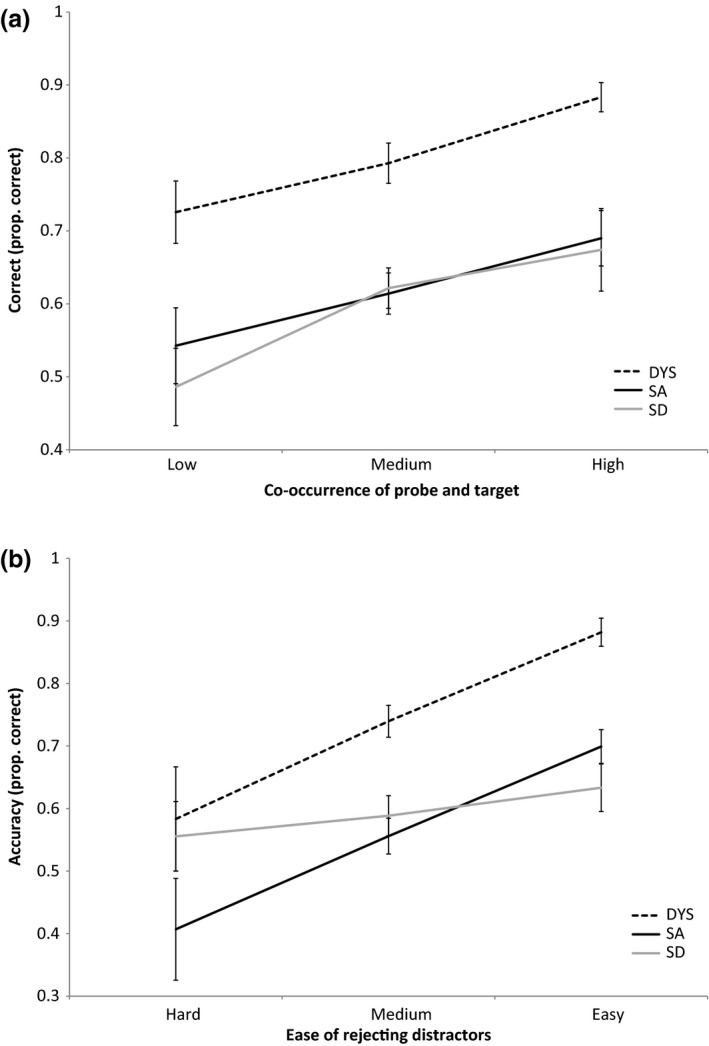
Effects of two aspects of trial difficulty on performance across patient groups. (a) Co‐occurrence of probe and target. (b) Ease of rejecting distractors. Ratings from 1 to 5: low/hard ≤2, medium >2 and <4, and high/easy ≥4. Error bars show standard error of mean.

Jefferies and Lambon Ralph (2006) collected ratings from healthy participants, who scored each item in the CCT from 1 to 5 in terms of these two aspects of difficulty. We used logistic regression to examine effects of these aspects of difficulty across groups: These models included group, rated difficulty, group x difficulty, CCT modality (word vs. picture task), patient ID, and familiarity. We further examined group effects by establishing which pairs of groups were significantly different, and which individual patients showed effects of difficulty. The results are shown in Figure 3.

We report the effects of group and the interaction between group and difficulty. For co‐occurrence of probe and target, there was no main effect of group or interaction with group (*W* ≤ 3.978, *p* ≥ .137). There was no difference in the effect of co‐occurrence between any of the group pairs (*W* ≤ 1.986, *p* ≥ .159). Each group individually showed a strong effect of this variable (*W* ≥ 5.148, *p* ≤ .023). However, for ease of rejecting distractors, there was a main effect of group, and an interaction between group and distractor strength: *W* ≥ 19.025, *p* < .001. There was a significant difference between SA and DYS: *W* = 3.844, *p* = .050, plus a highly significant difference between DYS and SD: *W* = 19.252, *p* < .001; and SA and SD: *W* = 9.880, *p* = .002. The effect of rejecting distractors was strong in the DYS group (*W* = 42.659, *p* < .001) and in SA patients (*W* = 40.022, *p* < .001), but absent in the SD group (*W* < 1).

### Manipulations of semantic control

Analyses of standard semantic assessments suggest that patients with executive dysfunction resemble SA cases and differ from SD patients. This supports the hypothesis that SA patients have difficulty controlling the retrieval of knowledge, as individuals with poor cognitive control but not selected to show language or semantic impairment have parallel deficits in semantic retrieval. In the next section, we directly test the hypothesis that semantic performance in patients with executive dysfunction is strongly influenced by the control demands of the task. We used the following manipulations: identifying close compared to distant semantic relationships (semantic distance task), dominant compared to subordinate meanings (ambiguity task), and strong compared to weak distractors (84‐item Synonym Task with distractors). We predicted equivalent effects of these manipulations in SA and DYS patients, as both groups are thought to have semantic problems that arise from insufficient control. Variable numbers of patients provided data on each task (*N* = 7–13), including seven SA cases published by Noonan *et al*. (2010; details below). A comparison group of SD cases was only available for the first of these tasks; therefore, we also describe results from the eight healthy controls reported by Noonan *et al*. (2010).

### Semantic distance

#### Rationale

Noonan *et al*. (2010) examined ‘nearest‐neighbour’ semantic judgements that manipulated the semantic distance of the probe and target (i.e., participants were asked to decide which word was most similar to the target). In the close condition, the probe and target shared many overlapping features, minimizing control demands. In the distant condition, the featural overlap between the probe and target was lower, making it harder to select the target and reject the distractors. Patients with semantic control deficits were expected to perform more poorly when the probe–target distance was greater, in line with previous findings (Noonan *et al*., 2010). Patients with degraded semantic representations, such as those with SD, were expected to show an attenuated difference between these conditions, as knowledge of the shared features that distinguish close and distant targets is thought to be eroded.

#### Methods

Participants were presented with a probe word and a target word with two distractors, in a 3AFC design. Words were written and also read aloud. There were 64 probes, and each probe was presented twice, once in the ‘close’ and once in the ‘distant’ condition (e.g., hat‐cap compared with hat‐stocking). In the close condition, the probe and target were from the same subcategory (clothing items that you wear on your head), in addition to their broader categorical similarities. In the distant condition, the probe and target were distantly related while sharing membership of the same broad semantic category (clothes). Distractors were targets drawn from different semantic categories. Testing was completed over two sessions, such that the same probe was not presented twice in the same session. We analysed the effect of semantic distance in 12 DYS patients, 13 SA patients (including seven reported by Noonan *et al*., 2010), eight SD patients (previously unreported data), and eight healthy controls (from Noonan *et al*., 2010).

#### Results

**Figure 4:**
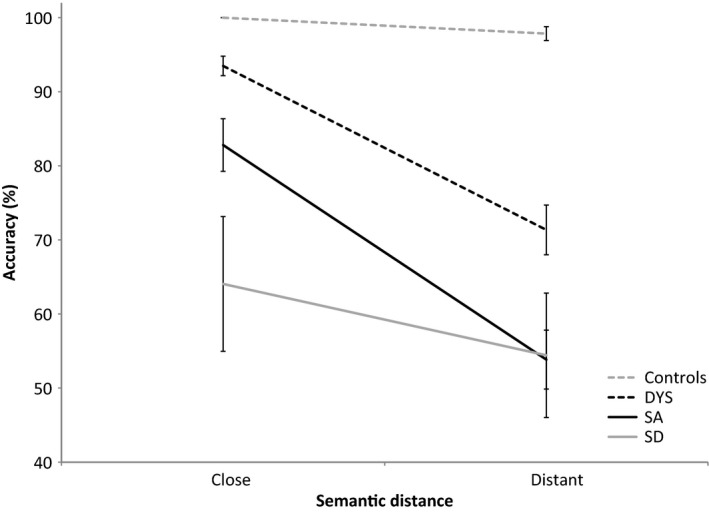
Effect of semantic distance on accuracy in DYS, SA, and SD patients in comparison with healthy controls. Error bars show standard error of mean.

The data are shown in Figure 4. We used an omnibus ANOVA to explore the effect of distance (close, distant) and group (controls, DYS, SA, SD). This found a significant effect of distance: *F* (1, 37) = 118.590, *p* < .001; group: *F* (3, 37) = 21.432, *p* < .001; and an interaction between distance and group: *F* (3, 37) = 17.436, *p* < .001.

Paired‐samples *t*‐tests confirmed a significant distance effect in all patient groups: DYS, *t* (11) = 7.608, *p* < .001, SA, *t* (12) = 9.475, *p* < .001, and SD, *t* (7) = 3.660, *p* = .008, with controls showing a marginal effect, *t* (7) = 2.308, *p* = .054. To explore relative effect of distance between groups, we computed ANOVAs for each pair of groups (Table 7). Controls showed the smallest effect of distance, followed by SD. SA and DYS were equivalent and more strongly influenced by distance. Thus, there was a significant interaction between distance and group in all cases except when DYS and SA patients were compared.

**Table 7:** Effect of semantic distance on performance across groups

	Distance	Group	Distance × group
Control vs. SA	*F* (1, 19) = 60.465, *p* < .001	*F* (1, 19) = 47.147, *p* < .001	*F* (1, 19) = 44.918, *p* < .001
Control vs. DYS	*F* (1, 18) = 43.702, *p* < .001	*F* (1, 18) = 39.714, *p* < .001	*F* (1, 18) = 29.604, *p* < .001
Control vs. SD	*F* (1, 14) = 17.726, *p* = .001	*F* (1, 14) = 41.922, *p* < .001	*F* (1, 14) = 7.378, *p* = .017
SA vs. DYS	*F* (1, 23) = 145.524, *p* < .001	*F* (1, 23) = 11.698, *p* = .002	n.s.
SA vs. SD	*F* (1, 19) = 76.134, *p* < .001	n.s.	*F* (1, 19) = 18.148, *p* = .001
DYS vs. SD	*F* (1, 18) = 58.096, *p* < .001	*F* (1, 18) = 18.896, *p* < .001	*F* (1, 18) = 8.359, *p* = .010

n.s. = not significant.

Each ANOVA was run on each pair of groups separately. *p* values reported if *p* < .1.

### Ambiguity

#### Rationale

Noonan *et al*. (2010) used polysemous words to test comprehension of dominant and subordinate meanings of words. Semantic control is thought to be required in selecting the less common interpretation of homonyms, and avoiding dominant but irrelevant interpretations (Rodd, Gaskell, & Marslen‐Wilson, 2004; Rodd *et al*., 2005; Zempleni, Renken, Hoeks, Hoogduin, & Stowe, 2007). Therefore, participants with disrupted semantic control should show greater difficulty comprehending less frequent meanings of ambiguous words. Additionally, when the relevant meaning is shaped by an external constraint, such as a sentence that cues the correct interpretation of the word, performance should increase. In contrast, a miscue that directs attention towards the irrelevant interpretation should impair performance by increasing activation of competitors.

#### Methods

An association matching task was used, where the participants selected which one of four words was related to the probe. All words were written and read aloud by the experimenter. The same probe was presented in the ‘dominant’ and ‘subordinate’ condition, but the target frequency was manipulated (e.g., pen‐pencil and pen‐pig). Interitem frequency (the frequency of the probe with the target) was higher for the dominant than the subordinate condition according to free association norms (Twilley, Dixon, Taylor, & Clark, 1994). The target words in dominant and subordinate conditions were matched for item frequency and imageability. The same distractors were used in both conditions. There were no‐cue, cue, and miscue conditions. In the cue condition, a sentence was given priming the appropriate meaning (e.g., ‘the labourers cleaned out the pen’ pen‐pig, or ‘he signed his name with a fountain pen’ pen‐pencil). In the miscue condition, the sentence cueing the opposite meaning of the word was used. Testing was carried out over six sessions. We analysed the ambiguity effect in nine DYS patients and 11 SA cases (seven of which were reported in Noonan *et al*., 2010). These patient groups were compared to eight healthy controls. No SD data were available for this task.

#### Results

**Figure 5:**
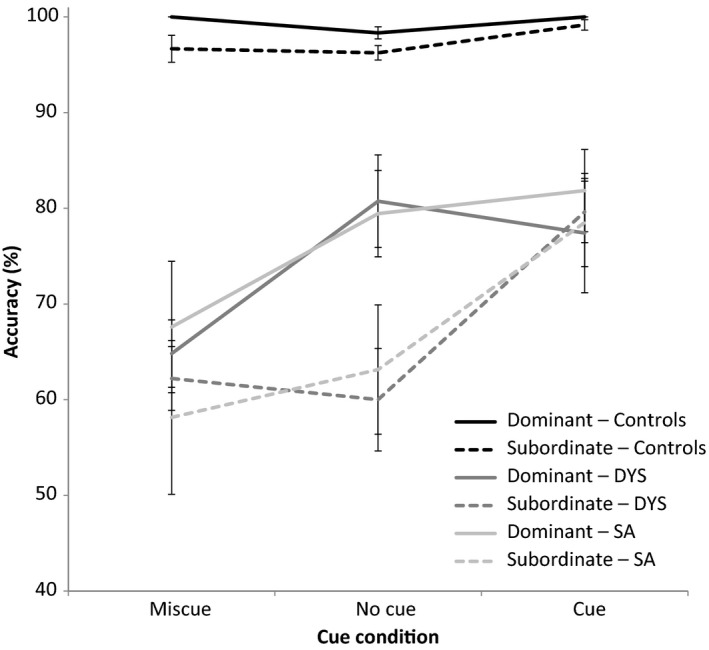
Performance on comprehension of ambiguous words in each cue condition. Error bars show standard error of mean.

In an omnibus 2‐by‐3‐by‐3 ANOVA examining ambiguity bias (dominant or non‐dominant interpretation of ambiguous word), cue (no cue, miscue, or cue), and group (control, SA, or DYS), there were main effects of cue: *F* (2, 50) = 58.931, *p* < .001; ambiguity bias: *F* (1, 25) = 32.372, *p* < .001; and group: *F* (2, 25) = 45.214, *p* < .001. All interactions were significant: cue x group: *F* (4, 50) = 16.428, *p* < .001; ambiguity bias x group: *F* (2, 25) = 7.158, *p* = .003; cue x ambiguity bias: *F* (2, 50) = 25.652, *p* < .001; and cue x ambiguity bias x group: *F* (4, 50) = 5.763, *p* = .001. This is shown in Figure 5.

To explore the results further, we collapsed the dominant/non‐dominant conditions and used corrected paired‐samples *t*‐tests to examine the effect of cueing in the patient groups. For DYS patients, there was a significant difference between no‐cue and cue trials: *t* (8) = 3.102, *p* = .045. There was no difference between no‐cue and miscue performance: *t* (8) = 2.525, *p* = .108. SA patients showed highly significant differences between both pairs of conditions: *t* (9) ≥ 6.402, *p* < .001. The cueing effect was stronger in SA than in DYS, as shown by an interaction between cue (miscue, cue, no cue) and group (SA, DYS): *F* (2, 36) = 5.995, *p* = .006.

We also collapsed across cue conditions to explore the interaction between patient group and ambiguity bias. There was a significant effect of ambiguity bias for both DYS, *t* (8) = 4.146, *p* = .003, and SA cases, *t* (17) = 7.738, *p* < .001. There was a near‐significant interaction between dominance (dominant, non‐dominant) and group (SA, DYS): *F* (1, 18) = 3.936, *p* = .063; DYS cases were somewhat less sensitive to miscuing of subordinate meanings.

### Synonym task with strong and weak distractors

#### Rationale

Noonan *et al*. (2010) examined the ability of SA patients to inhibit strongly associated distractor words when performing a synonym task, using tasks originally described in Samson, Connolly, and Humphreys (2007). Distractors are thought to create competition and increase control demands.

#### Methods

The design replicated Experiment 2 from Samson *et al*. (2007). Distractors shared a relationship with the probe (but were not a synonym and were not therefore a valid response). For example, the probe ‘piece’ was presented with the target ‘slice’ and the distractor ‘cake’ – there was also an unrelated distractor in a three‐alternative forced‐choice decision. This test consisted of 84 trials, 42 with weak targets and 42 with strong targets which were presented in a single block. We obtained data for 9 SA patients (seven from Noonan *et al*., 2010) and 12 DYS patients, to compare with eight healthy controls (from Noonan *et al*., 2010).

#### Results

**Figure 6:**
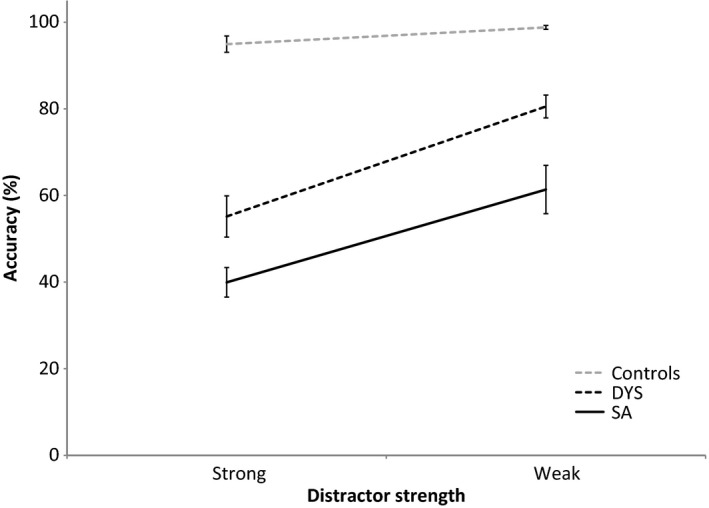
84‐item Synonym Task with distractors that are strongly or weakly related to the probe. Error bars show standard error of mean.

In an omnibus ANOVA, there were main effects of distractor strength: *F* (1, 26) = 85.301, *p* < .001; and group: *F* (2, 26) = 40.032, *p* < .001; plus an interaction: *F* (2, 26) = 12.503, *p* < .001. To understand this interaction further, we compared pairs of groups. There was a significant interaction between distractor strength and group in comparisons of DYS and controls, *F* (1, 18) = 32.923, *p* < .001, and SA and controls, *F* (1, 15) = 14.169, *p* = .002, but not in a comparison of DYS and SA patients (*F* < 1). Poorer performance for strong compared to weak distractor was seen in both patient groups (*t* ≥ 5.172, *p* < .001). This is shown in Figure 6.

### Manipulations of semantic control: Summary

On a range of tasks designed to manipulate semantic control demands, SA and DYS patients showed similar patterns. (1) Both patient groups showed effects of semantic distance, with poorer performance when attempting to match items that were further apart in semantic space (i.e., with fewer shared features) compared to those that were more similar. This effect contrasted with SD patients who showed similar effects to healthy controls (i.e., weaker effects of semantic distance). (2) In both DYS and SA groups, performance was relatively good when a dominant interpretation of an ambiguous word was required, and poorer when the subordinate meaning was probed, suggesting similar difficulties in inhibiting the more dominant meaning. Both groups showed positive effects of cues that reduced the requirement to generate internal constraints on semantic retrieval. (3) Both DYS and SA patients showed equivalent effects of distractor strength, with poorer performance when a highly salient but irrelevant distractor was present. All these findings suggest the two patient groups have parallel disruption of controlled semantic access and a deficit in the flexible retrieval of conceptual information.

## Discussion

The CSC framework (Jefferies, 2013; Lambon Ralph *et al*., 2017; Rogers *et al*., 2015) suggests that the successful retrieval and application of conceptual information to drive appropriate thoughts and behaviour requires interaction between brain regions that store knowledge and those that support the controlled access to such information. In patients with SA, it has been argued that semantic control mechanisms are disrupted, giving rise to a pattern of performance that reflects intact semantic representations but impaired controlled retrieval (Jefferies, 2013; Jefferies & Lambon Ralph, 2006; Noonan *et al*., 2010). This pattern contrasts with SD, in which transmodal conceptual information becomes degraded (Bozeat *et al*., 2000; Jefferies *et al*., 2010; Lambon Ralph & Patterson, 2003; Lambon Ralph *et al*., 2010). To test this hypothesis, patients with executive impairments (DYS) – not selected to show any semantic deficits – were tested and compared with SA patients and SD patients. It was not possible to differentiate the groups on the basis of test scores. Strikingly, the DYS and SA patients showed largely parallel performance on a range of semantic tasks. The similarity between these two heterogeneous groups, selected in contrasting ways, provides further information about the way in which executive processes support the appropriate and flexible use of semantic concepts. The findings also suggest that deficits that have been well characterized in SA may be relatively widespread in patients with varying aetiologies who have executive deficits.

There were at least eight areas of similarity between the DYS and SA groups shown in this study:

1. *Parallel deficits in semantic and executive tasks*: SA patients were more impaired than DYS cases on both semantic and executive tasks, but the groups showed an equivalent relationship between these domains. Patients with executive dysfunction, not selected to show semantic deficits, nonetheless showed impairment on our standard semantic battery. Similarly, SA patients, selected to show multimodal semantic impairments, had executive deficits correlating with semantic performance. Our results are compatible with the proposal that executive control allows semantic knowledge to be applied in a task‐appropriate way, and are consistent with the observation that the brain regions that participate in semantic control and in the multidemand executive network are highly overlapping (Noonan *et al*., 2013; Whitney *et al*., 2012).
2. *Performance within and across semantic tasks*: SD patients show a high degree of consistency in their performance on the same items across tasks: This is thought to reflect the degradation of central semantic representations – that is, knowledge that is still available in one task strongly predicts whether conceptual representations will be available to support performance in other tasks (Bozeat *et al*., 2000; Jefferies & Lambon Ralph, 2006). In contrast, both DYS and SA showed considerably less consistency between tasks with differing control demands.
3. *Familiarity/frequency*: Patients with executive deficits did not show the typical processing advantage for high‐frequency/familiar words. As high‐frequency words are encountered in a wider range of situations than low‐frequency words, only a subset of their associations and features are relevant in any given context (Adelman, Brown, & Quesada, 2006; Almaghyuli *et al*., 2012; Hoffman, Jefferies, & Lambon Ralph, 2011; Hoffman, Rogers, *et al*., 2011). This increases the requirement to ‘shape’ semantic retrieval to suit the context being probed and may have removed the positive effects of concept familiarity and lexical frequency across a range of tasks (i.e., 96‐item Synonym Judgement Task; CCT for words and pictures; Environmental Sounds Task) in the SA and DYS groups. In contrast, these variables have a strong positive effect on comprehension in SD patients – presumably because frequently encountered items form stronger conceptual representations that are more resistant to damage (Bozeat *et al*., 2000; Funnell, 1995; Jefferies & Lambon Ralph, 2006; Jefferies *et al*., 2009).
4. *Trial difficulty*: In judgements of semantic association, we examined different facets of rated trial difficulty: the frequency of co‐occurrence of the probe and target, and the ease of rejecting the distractors. Probe–target co‐occurrence affected all patient groups (SD, SA, DYS), presumably because frequently encountered associations are encoded more strongly in the semantic store (and are therefore more resistant to damage in SD, but also retrieved more easily in SA and DYS cases). In contrast, the ease of rejecting the distractors affected SA and DYS but not SD patients: Only individuals with semantic control deficits were vulnerable to the degree of competition with the target, as they had difficulty selecting relevant information.
5. *Semantic distance*: In the nearest‐neighbour task, difficulty was manipulated by comparing close and more distant probe and target pairs (Noonan *et al*., 2010). In the easy condition (e.g., ship and yacht), the probe and target shared many features and it was relatively easy to select the target from amongst distractors. In the difficult condition, the probe and target shared few features (e.g., ship and van), making the target more difficult to select. A strong effect of semantic distance was found in SA and DYS patients, but this was attenuated in SD.
6. *Ambiguity*: Homonyms with dominant and subordinate meanings were used to explore the comprehension of ambiguous words (Noonan *et al*., 2010) in SA and DYS patients. Dominant meanings are thought to be retrieved relatively automatically, in the absence of control. When a non‐dominant interpretation is required, however, strong but irrelevant associations must be inhibited to allow the weaker interpretation to come to the fore. The SA and DYS cases showed parallel deficits in the retrieval of non‐dominant interpretations, consistent with difficulty in constraining semantic activation in both groups.
7. *Cueing*: The ambiguity task was combined with a cueing manipulation, in which a sentence was presented before each trial either to cue the correct interpretation of the homonym or to miscue the incorrect meaning (Noonan *et al*., 2010). Both DYS and SA patients showed positive effects of cues, consistent with the view that executive–semantic mechanisms are required to shape retrieval to suit the meaning being probed in the absence of external constraints.
8. *Distractor strength*: We examined synonym judgement with and without strong distractors. SA and DYS patients had similar difficulty inhibiting irrelevant but related distractors, suggesting impaired executive–semantic processing.

In all of these tasks, patients with executive dysfunction had similar semantic deficits to those seen in SA. This lends support to the hypothesis that domain‐general executive processes interact with semantic representations to support controlled aspects of semantic cognition, and provides further evidence for the proposal that SA cases have a semantic deficit that reflects poor control over conceptual retrieval. While there are some regions implicated in semantic and not domain‐general executive control, such as pMTG and anterior ventral IFG (e.g., Badre *et al*., 2005; Davey, Cornelissen, *et al*., 2015, 2016; Humphreys & Lambon Ralph, 2015; Noonan *et al*., 2013), controlled semantic processing is supported by multidemand as well as semantic regions (Humphreys & Lambon Ralph, 2015; Noonan *et al*., 2013; Whitney *et al*., 2012). Indeed, although the function of these regions appears to be at least partially distinct, recent network (path) analyses of white‐matter DTI and resting‐state fMRI data indicate that they form a ‘single functional module’ arising from their physical white‐matter connections (Jung, Cloutman, Binney, & Lambon Ralph, 2016). For example, although pMTG is not generally considered to be part of the multidemand network, it has strong white‐matter connectivity to lateral prefrontal regions and intraparietal sulcus (Binney, Parker, & Lambon Ralph, 2012; Jung *et al*., 2016). Moreover, regions implicated in semantic control (pMTG; anterior IFG) lie between the multidemand network and the anterior temporal lobe implicated in the representation of heteromodal conceptual knowledge, in terms of both their location on the cortical surface and intrinsic functional connectivity (Davey *et al*., 2016): If semantic control regions allow orthogonal representations of task context and conceptual knowledge to be integrated, these regions may not function normally in the face of significant disruption to the executive network. Given the relatively large and perfuse lesions which can occur after stroke or brain injury, and the limited availability of lesion data in this study, further research employing fMRI during the process of semantic retrieval in DYS and SA cases is needed to examine this hypothesis.

In conclusion, we show for the first time that patients with executive dysfunction have deficits in semantic cognition similar to those observed in patients with semantic aphasia. As a result of the underlying executive impairments, both SA and DYS patients find it difficult to manipulate and gate semantic information in order to generate context‐, task‐, and time‐appropriate behaviours. These results support the CSC framework which proposes that semantic cognition involves the interplay of conceptual knowledge and control processes that guide retrieval.

## Supporting information


**Figure S1.** Effects of familiarity on accuracy on the Cambridge Semantic Task.
**Table S1.** Performance across background tests – SA and SD patients.
**Table S2.** Factor analysis by group.
**Table S3.** Scores on the Cambridge Semantic Battery according to familiarity.
**Appendix S1**. Comparing Executive and Semantic performance in DYS and SA patients.
