# Supplementary material for: The contribution of executive control to semantic cognition: Convergent evidence from semantic aphasia and executive dysfunction
Source: J Neuropsychol. 2018 Jan 3;12(2):312–40. doi: 10.1111/jnp.12142 (PMC6001665; doi:10.1111/jnp.12142)
Supplement: Supplementary file 1 — Figure S1. Effects of familiarity on accuracy on the Cambridge Semantic Task. Table S1. Performance across background tests – SA and SD patients. Table S2. Factor analysis by group. Table S3. Scores on the Cambridge Semantic Battery according to familiarity. Appendix S1. Comparing Executive and Semantic performance in DYS and SA patients. [file JNP-12-312-s001.docx]

Table S1: Performance across background tests – SA and SD patients

|  | Brixton | Ravens | digit span | backwards digit span | letter fluency (F,A,S) | WPM | naming | CCTw | CCTp | S-P env sounds | W-P env sounds | 96-item synonym | Low imageability | Medium imageability | High imageability | Low frequency | High frequency |
| --- | --- | --- | --- | --- | --- | --- | --- | --- | --- | --- | --- | --- | --- | --- | --- | --- | --- |
| SA patients | | | | | | | | | | | | | | | | | |
| HN | 28 | 20*** | 6 | 2** | 19 | 50*** | 50*** | 54* | 54 | 36 | 16*** | 70*** | 27* | 31 | 32 | 43 | 47 |
| EW | 33 | 30 | 4*** | 2** | 20 | 57*** | 45*** | 48*** | 45** | 22*** | 45** | 76*** | 20*** | 25*** | 31 | 38*** | 38*** |
| JD | 28 | 30 | 5* | 2** | 5 | 64 | 49*** | 38*** | 38*** | 23*** | 46* | 73*** | 20*** | 27*** | 26*** | 40*** | 33*** |
| SC | 25 | 22*** | 6 | 2** | 24 | 59*** | 28*** | 56 | 47** | 32* | 41*** | 71*** | 14*** | 28*** | 29*** | 35*** | 36*** |
| ME | 11 | 13*** | 6 | 3* | 14 | 50*** | 4*** | 34*** | 13*** | 33* | 40*** | 81*** | 20*** | 30* | 30** | 38*** | 42*** |
| GH | 18 | 32 | 2*** | 0*** | 2 | 60*** | 19*** | 29*** | 45** | NT | NT | 71*** | 17*** | 25*** | 29*** | 40*** | 31*** |
| NY | 34 | 26* | 3*** | 2** | 5 | 60*** | 51*** | 39*** | 36*** | 28** | 40*** | 69*** | 15*** | 26*** | 28*** | 36*** | 33*** |
| PG | 26 | 23*** | 6 | 2** | 2 | 58*** | 44*** | 40*** | 44** | 33* | 47 | 69*** | 19*** | 21*** | 29*** | 36*** | 33*** |
| JM | NT | 14*** | 3*** | 2** | 1 | 53*** | 30*** | 37*** | 37*** | 24*** | 43*** | 27*** | 8*** | 13*** | 10*** | 14*** | 13*** |
| MS | 16 | 5*** | 0*** | NT | 0 | 46*** | 0*** | 42*** | 37*** | 28** | 37*** | TA | - | - | - | - | - |
| KH | 7 | 12*** | 4*** | 2** | 0 | 54*** | 29*** | 41*** | 46** | 30** | 44*** | 61*** | 14*** | 21*** | 26*** | 27*** | 34*** |
| KA | 6 | 12*** | 0*** | NT | NT | 26*** | NT | 36*** | 46** | 22*** | 21*** | 60*** | 19*** | 16*** | 25*** | 29*** | 31*** |
| BB | 23 | 24** | 5* | 0*** | 0 | 54*** | 9*** | 30*** | 38*** | 26*** | 33*** | 63*** | 21*** | 15*** | 27*** | 31*** | 32*** |
| DB | 31 | 31 | 4*** | NT | 1 | 46*** | 39*** | 46*** | 51* | 21*** | 36*** | 54*** | 12*** | 16*** | 26*** | 25*** | 29*** |
| LS | 14 | 16*** | 4*** | 1** | 8 | 37*** | 5*** | 16*** | 15*** | 27** | 35*** | 47*** | 13*** | 13*** | 21*** | 22*** | 25*** |
| EC | 24 | 12*** | 0*** | 0*** | 0 | 40*** | 1*** | 20*** | 32*** | NT | NT | 41*** | 14*** | 10*** | 17*** | 21*** | 20*** |
| KS | 29 | 31 | 8 | 4 | 19 | 46*** | 21*** | 42*** | 44** | NT | NT | 81*** | 19*** | 30* | 32 | 37*** | 44* |
| BK | 26 | 24** | 5* | 2** | NT | 63 | 50*** | 53** | 56 | NT | NT | 74*** | 17*** | 26*** | 31 | 37*** | 37*** |
| PH | 7 | 27* | 4*** | 2** | NT | 62* | 61 | 43*** | 44** | NT | NT | 58*** | 11*** | 21*** | 26*** | 25*** | 33*** |
| EG | 21 | 31 | 0*** | NT | NT | 63 | 1*** | 39*** | 31*** | NT | NT | 57*** | 13*** | 22*** | 22*** | 30*** | 27*** |
| MP | 25 | 33 | 0*** | NT | NT | NT | NT | 39*** | 51* | 26*** | 24*** | 62*** | 15*** | 18*** | 29*** | 31*** | 31*** |
| CH | 26 | 25** | 6 | 4 | NT | 53*** | NT | NT | NT | NT | 39*** | 81*** | 21*** | 30* | 30** | 41*** | 40*** |
| MD | 21 | 34 | 4*** | 2** | NT | 60*** | 44*** | 51** | 32*** | 24*** | NT | 85*** | 23*** | 30* | 32 | 45* | 40*** |
| MJ | 22 | 16*** | 2*** | NT | NT | 37*** | 28*** | 29*** | 37*** | 20*** | 27*** | 61*** | 14*** | 19*** | 28*** | 31*** | 30*** |
|  | | | | | | | | | | | | | | | | | |
| SD patients | | | | | | | | | | | | | | | | | |
| JP |  |  | 6 | 5 | 27 | 64 | 59 | 62 | 61 | 23*** | 43*** |  |  |  |  |  |  |
| WM |  |  | 8 | 7 | 29 | 63 | 57* | 52* | 56 | 42 | 48 |  |  |  |  |  |  |
| SL |  |  | 6 | 3* | 31 | 60*** | 45*** | 34*** | 52 | 29** | 38*** |  |  |  |  |  |  |
| AT |  |  | 8 | 5 | 20 | 58*** | 43*** | 37*** | 47** | 20*** | 42*** |  |  |  |  |  |  |
| JC |  |  | 8 | 4 | 23 | 57*** | 17*** | 43*** | 51* | 21*** | 39*** |  |  |  |  |  |  |
| DS |  |  | 6 | 4 | 7 | 58*** | 17*** | 44*** | 43** | 23*** | 40*** |  |  |  |  |  |  |
| DC |  |  | 7 | 2** | 16 | 36*** | 11*** | 18*** | 31*** | 15*** | 25*** |  |  |  |  |  |  |
| JH |  |  | 6 | 5 | 19 | 18*** | 6*** |  | 30*** | 14*** | 20*** |  |  |  |  |  |  |
| JW |  |  | 5* | 5 | NT | 23*** | 9*** |  | 22*** | 14*** | 23*** |  |  |  |  |  |  |
| IF |  |  | 5* | 5 | 16 | 18*** | 1*** | 10*** | 19*** | 15*** | 13*** |  |  |  |  |  |  |
| AN |  |  |  |  |  |  |  |  |  |  |  | 81*** | 25*** | 25*** | 31 | 35*** | 46 |
| WM |  |  |  |  |  |  |  |  |  |  |  | 81*** | 24*** | 26*** | 31 | 34*** | 47 |
| SJ |  |  |  |  |  |  |  |  |  |  |  | 79*** | 19*** | 29*** | 31 | 35*** | 44* |
| EK |  |  |  |  |  |  |  |  |  |  |  | 70*** | 18*** | 24*** | 28*** | 29*** | 41* |
| LS |  |  |  |  |  |  |  |  |  |  |  | 64*** | 15*** | 25*** | 24*** | 28*** | 36*** |
| GE |  |  |  |  |  |  |  |  |  |  |  | 63*** | 19*** | 22*** | 22*** | 17*** | 46 |
| GT |  |  |  |  |  |  |  |  |  |  |  | 64*** | 16*** | 24*** | 24*** | 27*** | 37*** |
| PD |  |  |  |  |  |  |  |  |  |  |  | 59*** | 15*** | 23*** | 21*** | 22*** | 37*** |
| KI |  |  |  |  |  |  |  |  |  |  |  | 59*** | 17*** | 18*** | 24*** | 21*** | 38*** |
| MK |  |  |  |  |  |  |  |  |  |  |  | 30*** | 4*** | 9*** | 17*** | 6*** | 24*** |
| ATe |  |  |  |  |  |  |  |  |  |  |  | 26*** | 1*** | 10*** | 15*** | 5*** | 21*** |

* p < .05, ** p < .01, *** p < .001 two-tailed probability using the “Singlims” procedure (Crawford & Garthwaite, 2002), which uses a modified t-statistic to examine whether an individual is significantly below a control group, taking into account group size and standard deviation. TA = test abandoned. NT = not tested. Brixton = Brixton Spatial Rule Assessment (Burgess & Shallice, 1997), Ravens = Ravens Coloured Progressive Matrices ([Raven, 1962](#_ENREF_1)), digit span and backwards digit span ([Wechsler, 1987](#_ENREF_2)), WPM = word-picture matching, naming, CCTw = camel and cactus words, CCTp = camel and cactus pictures. All four tasks from the Cambridge Semantic Battery (Bozeat et al., 2000). S-P env sounds = sound-picture matching; W-P env sounds = word-picture matching, from the environmental sounds task (Bozeat et al., 2000). 96-item synonym and the subscores according to imageability/frequency (Jefferies et al., 2009).

Table S2: Factor analysis by group

| Patient | Group | Semantic Factor | Executive Factor |
| --- | --- | --- | --- |
| 1 | DYS | -1.78370 | -1.96476 |
| 2 | DYS | -.70654 | -1.82189 |
| 3 | DYS | 1.94023 | .28228 |
| 4 | DYS | -.27981 | .95330 |
| 5 | DYS | -.46479 | -.24321 |
| 6 | DYS | -.08112 | .14074 |
| 7 | DYS | .54585 | .61712 |
| 8 | DYS | .03159 | -.09856 |
| 9 | DYS | -.90757 | .23540 |
| 10 | DYS | 1.23481 | -.19588 |
| 11 | DYS | -.26220 | .85642 |
| 12 | DYS | .73325 | 1.23904 |
|  | | | |
| HN | SA | 1.01562 | .26181 |
| EW | SA | .89524 | 1.25245 |
| JD | SA | .48077 | .94320 |
| SC | SA | 1.20131 | .21254 |
| ME | SA | -.58347 | -1.26660 |
| GH | SA | .16176 | .46098 |
| NY | SA | .25645 | 1.04175 |
| PG | SA | .44511 | .34253 |
| JM | SA | -.92195 | -1.87881 |
| MS | SA | - | -1.50247 |
| KH | SA | .23943 | -1.58214 |
| KA | SA | -.82289 | -1.64399 |
| BB | SA | -.34313 | .22512 |
| DB | SA | .16395 | 1.19689 |
| LS | SA | -2.34609 | -.87664 |
| EC | SA | -1.78485 | -.53070 |
| KS | SA | - | 1.07319 |
| BK | SA | 1.54428 | .41067 |
| PH | SA | .46163 | -.56006 |
| EG | SA | -.04652 | .57840 |
| MP | SA | - | .96207 |
| CH | SA | - | .47881 |
| MD | SA | .95167 | .78281 |
| MJ | SA | -.96830 | -.38184 |

Factor analysis scores for each group separately. Factors are presented as coefficients using Varimax rotation, extracting a fixed number of factors (one) per analysis.

Table S3: Scores on the Cambridge Semantic Battery according to familiarity

|  | Low familiarity | | High familiarity | |
| --- | --- | --- | --- | --- |
| DYS - CCTp | 81.3 | (2.5) | 80.4 | (1.7) |
| DYS - CCTw | 73.4 | (2.8) | 82.6 | (1.7) |
| DYS - WPM | 94.8 | (1.4) | 94.8 | (1.0) |
| DYS - naming | 81.9 | (2.7) | 86.0 | (1.7) |
| DYS - W-P | 92.6 | (1.8) | 97.6 | (0.8) |
| DYS - S-P | 57.4 | (3.5) | 82.5 | (2.0) |
| SA - CCTp | 64.9 | (2.2) | 61.1 | (1.6) |
| SA - CCTw | 62.4 | (2.3) | 60.8 | (1.6) |
| SA - WPM | 80.5 | (1.8) | 79.7 | (1.3) |
| SA - naming | 39.5 | (2.4) | 49.0 | (1.7) |
| SA - W-P | 74.8 | (2.8) | 76.7 | (2.0) |
| SA - S-P | 54.6 | (3.2) | 56.2 | (2.4) |
| SD - CCTp | 53.8 | (3.4) | 67.7 | (2.3) |
| SD - CCTw | 51.8 | (3.9) | 61.0 | (2.6) |
| SD - WPM | 61.9 | (3.4) | 75.6 | (2.1) |
| SD - naming | 28.1 | (3.1) | 47.9 | (2.4) |
| SD - W-P | 46.3 | (4.3) | 75.8 | (2.7) |
| SD - S-P | 31.6 | (4.0) | 50.0 | (3.2) |

Scores shown as percentages, mean (SE). WPM = word-picture matching, naming, CCTw = camel and cactus words, CCTp = camel and cactus pictures. All four tasks from the Cambridge Semantic Battery (Bozeat et al., 2000). S-P = sound-picture matching; W-P = word-picture matching, from the environmental sounds task (Bozeat et al., 2000). For the Cambridge Semantic Battery, low familiarity < 2.5 out of 5, high familiarity ≥ 2.5 out of 5. For the environmental sounds task, low familiarity < 3 out of 6, high familiarity ≥ 3 out of 6.

Figure S1: Effects of familiarity on accuracy on the Cambridge Semantic Task (averaged performance on the four tasks from the Cambridge Semantic Battery and two tasks from the Environmental sounds task (both Bozeat et al., 2000)).

Supplementary Analysis 1: Comparing Executive and Semantic performance in DYS and SA patients

Factor analysis was used to extract a single factor across multiple tasks which tap the same concept (e.g., semantic, executive). We originally ran this analysis tasks which did not involve spoken output. However, for completeness we re-ran the analysis including all executive and semantic tasks where we had the majority of patient scores. As in the original analysis, the factor analysis was initially run on each group separately, before comparing normalised scores across groups. The ‘executive’ factor included Brixton, Raven’s Coloured Progressive Matrices, backwards digit span, and letter fluency. The ‘semantic’ factor included WPM, CCTw, CCTp, the 96-item synonyms and naming.

Here there was a main effect of group (F(1,24) = 8.146, p = .006), and executive factor (F(1,24) = 20.995, p < .001), as well as a significant interaction: F(1,24) = 9.958, p = .004. This is shown in Figure S1.

Figure S1: The correlation between Executive and Semantic performance for DYS and SA patients.
